# 17-Aminogeldanamycin Inhibits Constitutive Nuclear Factor-Kappa B (NF-κB) Activity in Patient-Derived Melanoma Cell Lines

**DOI:** 10.3390/ijms21113749

**Published:** 2020-05-26

**Authors:** Mariusz L. Hartman, Magdalena Rogut, Aleksandra Mielczarek-Lewandowska, Michal Wozniak, Malgorzata Czyz

**Affiliations:** Department of Molecular Biology of Cancer, Medical University of Lodz, 92-215 Lodz, Poland; magdalena.rogut@stud.umed.lodz.pl (M.R.); amielczarek1991@o2.pl (A.M.-L.); michal.wozniak@umed.lodz.pl (M.W.); malgorzata.czyz@umed.lodz.pl (M.C.)

**Keywords:** 17-aminogeldanamycin, BCL-X_L_, BRAF, cyclin D1, HSP90 inhibitor, IL-8, melanoma, NRAS, p65/NF-κB, VEGF

## Abstract

Melanoma remains incurable skin cancer, and targeting heat shock protein 90 (HSP90) is a promising therapeutic approach. In this study, we investigate the effect of 17-aminogeldanamycin, a potent HSP90 inhibitor, on nuclear factor-kappa B (NF-κB) activity in BRAF^V600E^ and NRAS^Q61R^ patient-derived melanoma cell lines. We performed time-lapse microscopy and flow cytometry to monitor changes in cell confluence and viability. The NF-κB activity was determined by immunodetection of phospho-p65 and assessment of expression of NF-κB-dependent genes by quantitative real-time polymerase chain reaction (qRT-PCR), Western blotting, and enzyme-linked immunosorbent assay (ELISA). Constitutive activity of p65/NF-κB was evident in all melanoma cell lines. Differences in its level might be associated with genetic alterations in *CHUK*, *IL1B*, *MAP3K14*, *NFKBIE*, *RIPK1*, and *TLR4*, while differences in transcript levels of NF-κB-inducible genes revealed by PCR array might result from the contribution of other regulatory mechanisms. 17-Aminogeldanamycin markedly diminished the level of phospho-p65, but the total p65 protein level was unaltered, indicating that 17-aminogeldanamycin inhibited activation of p65/NF-κB. This conclusion was supported by significantly reduced expression of selected NF-κB-dependent genes: cyclin D1 (*CCND1)*, C-X-C motif chemokine ligand 8 (*CXCL8*), and vascular endothelial growth factor (*VEGF*), as shown at transcript and protein levels, as well as secretion of IL-8 and VEGF. Our study indicates that 17-aminogeldanamycin can be used for efficient inhibition of NF-κB activity and the simultaneous diminution of IL-8 and VEGF levels in the extracellular milieu of melanoma.

## 1. Introduction

Melanoma is one of the most aggressive human cancers, posing a serious clinical problem worldwide due to an increasing incidence and limited therapeutic regimens for the advanced stage of the disease [1,2]. The majority of melanomas harbor mutations in either *BRAF* or *RAS* or *NF1* that result in hyperactivation of the RAS/RAF/MEK/ERK (hereafter MAPK) signaling pathway [3]. Although this has propelled the development of targeted therapeutics, the treatment with BRAF^V600^ inhibitors such as vemurafenib [4,5] or dabrafenib [6,7] almost inevitably results in drug-resistant disease despite an initially potent response [8,9]. The combination of BRAF and MEK inhibitors has been proven to be advantageous compared to monotherapy [10,11], and a novel drug combination of encorafenib (inhibitor of BRAF^mut^) and binimetinib (inhibitor of MEK1/2) has been approved for the treatment of patients with unresectable or metastatic melanoma [12]. However, available preclinical and clinical observations indicate that drug resistance and disease progression still occur despite the synergistic action of BRAF and MEK inhibitors [13,14], suggesting that vertical targeting of the MAPK signaling pathway may be insufficient to achieve a durable response. In addition, 41–81% melanoma patients do not respond to immunotherapy, which is another treatment option currently used in the clinics [14]. This indicates that alternative or complementary drug targets are needed.

A heat shock protein 90 (HSP90) is upregulated in melanoma, and its level increases with disease progression [15]. HSP90 is required for folding of a number of oncoproteins relevant to melanoma, including BRAF^V600E^ but not a wild-type variant of BRAF, and components of the phosphatidylinositol 3-kinase (PI3K)/AKT, wingless-type (WNT)/β-catenin, unfolded protein response (UPR), and nuclear factor-kappa B (NF-κB) signaling pathways [16,17,18]. As a consequence, several inhibitors of HSP90 have been investigated in melanoma, demonstrating that these agents can be effective either as a single or complementary therapeutic strategy [18,19]. We have recently shown that 17-aminogeldanamycin, an inhibitor of HSP90, is more potent against melanoma cells than its parent compound, geldanamycin [20,21]. As reported for N-terminal HSP90 inhibitors, 17-aminogeldanamycin induces a compensatory response involving the upregulation of *HSP70* expression, but this effect is transient and followed by the induction of cell death [21]. In addition, 17-aminogeldanamycin acts cooperatively with either vemurafenib or trametinib in the induction of apoptosis in BRAF^V600E^ and NRAS^Q61R^ melanoma cells [21]. The effect of 17-aminogeldanamycin on the NF-κB signaling has not been investigated so far.

To evaluate the effects of 17-aminogeldanamycin on the p65/NF-κB program in melanoma, we used six patient-derived cell lines, representing different genetic subtypes, either BRAF^V600E^ (DMBC11, DMBC12, DMBC21, DMBC28, and DMBC29) or NRAS^Q61R^ (DMBC22) subtypes. These cell lines have already been extensively characterized, considering cell morphology, activities of melanoma-associated signaling pathways, and genetic alterations [21,22,23,24,25,26,27].

## 2. Results

### 2.1. Patient-Derived Melanoma Cell Lines Differently Execute the p65/NF-κB-Dependent Program

**Figure 1:**
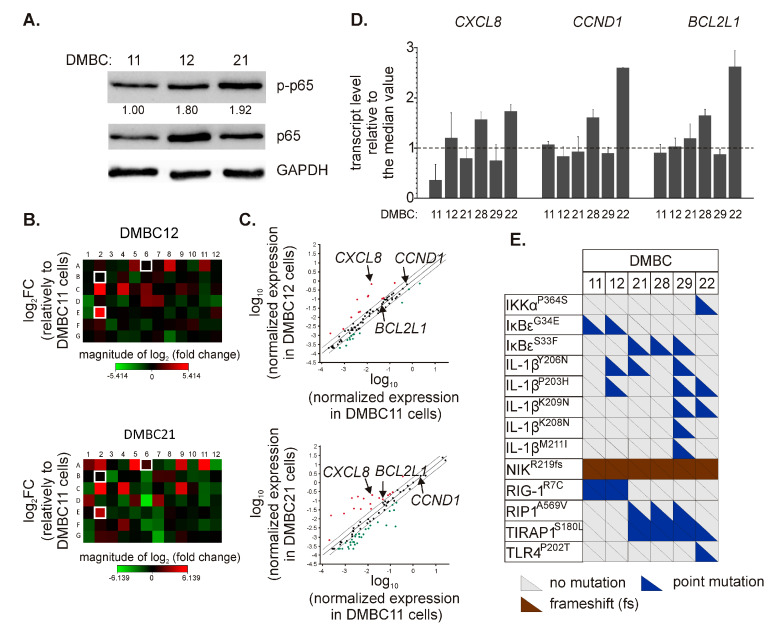
Diverse execution of nuclear factor-kappa B (NF-κB)-dependent program in melanoma cell lines. (**A**) Levels of phosphorylated (p-p65) and total p65 were determined by Western blotting. Glyceraldehyde-3-phosphate dehydrogenase (GAPDH) was used as a loading control. The mean relative level of p-p65 *versus* GAPDH is shown (*n* = 3). (**B**) Heatmaps were prepared to visualize differentially expressed NF-κB-dependent genes. The relative mRNA levels in DMBC12 and DMBC21 cells were obtained by normalization to their levels in DMBC11 cells. Each fold-change (FC) value was log_2_-transformed. The array layout is shown in Table S1. The genes selected for further experiments are white framed. (**C**) Scatter plots were obtained by log_10_ normalization of gene expression. Upper and lower bounds were set for a 2-fold change in mRNA levels. Upregulated genes are shown in red, and downregulated genes are shown in green. Detailed data are shown in Table 1. (**D**) The basal transcript levels of C-X-C motif chemokine ligand 8 (*CXCL8*), cyclin D1 (*CCND1*), and BCL-2-like 1 (*BCL2L1*) in different melanoma cell lines of both BRAF^V600E^ and NRAS^Q61R^ subtypes were assessed by quantitative real-time polymerase chain reaction (qRT-PCR) and expressed relative to the median values in all six cell lines. (**E**) Color-coded diagram of mutations in genes assigned to the NF-κB signaling (based on the KEGG PATHWAY database). Only indels and point mutations with damaging PolyPhen-2-based predictions are shown. Each triangle represents a single allele. Detailed data and non-mutated genes are included in Table S2.

Three cell lines, DMBC11, DMBC12, and DMBC21, were initially chosen to investigate the activity of NF-κB. As shown in Figure 1A, these cell lines slightly differed in the levels of p65 and its active form, p-p65, with the DMBC11 cell line exerting the lowest level. Next, we used a Profiler PCR array to more extensively analyze the p65/NF-κB-dependent program by comparing the expression of 84 NF-κB target genes. Gene expression was calculated relative to DMBC11 cells. We found a number of genes downregulated in DMBC21 cells compared with the DMBC11 cell line (Figure 1B). When the cut-off was set as a 2-fold change, 13 and 30 genes were downregulated in DMBC12 and DMBC21 cells, respectively (Figure 1C and Table 1). DMBC21 cells largely differed from DMBC11 cell line, and 7 out of 30 downregulated genes exceeded a 5-fold lower level than in DMBC11 cells, including *IL1A*, *TNFRSF1B*, *CCL22*, *CCR5*, *PLAU*, *CD80*, and *PTGS2* (Figure 1C and Table 1). In turn, 12 and 18 genes were upregulated in DMBC12 and DMBC21 cells, respectively, compared with DMBC11 cells (Figure 1C and Table 1). Genes encoding chemokines and interleukins (*CXCL1*, *CXCL2*, *CXCL8*, *CCL2*, and *IL1B*), antiapoptotic proteins (*BCL2A1* and *BCL2L1*) and coagulation factor VIII (*F8*) were among those differentially expressed. In addition, higher expression of *C3*, *AGT,* and *ICAM1* was found in DMBC21 cells than in DMBC11 cells (Figure 1C and Table 1).

For the following experiments, we have chosen three representative genes, C-X-C motif chemokine ligand 8 (*CXCL8*), cyclin D1 (*CCND1*), and BCL-2-like 1 (*BCL2L1*), as they (i) encode proteins involved in different cellular processes, i.e., invasion/migration and angiogenesis, proliferation and survival, respectively, and (ii) are characterized as relevant to melanoma. We did not choose *BCL2A1*, although its basal expression was highly upregulated in DMBC21 compared with DMBC11 cells, as *BCL2A1* is also a target of microphthalmia-associated transcription factor (MITF), which is a melanoma-specific protein [28]. In agreement, we have previously demonstrated substantial upregulation of *BCL2A1* expression in MITF^high^ melanoma cells [29,30,31,32].

We assessed mRNA levels of selected genes in six melanoma cell lines assigned to the BRAF^V600E^ or NRAS^Q61R^ subtype. The expression of *CCND1* and *BCL2L1* at the transcript level was very close, as shown in relation to the median values, except for their expression in DMBC28 and DMBC22 that was substantially higher (Figure 1D). *CXCL8* mRNA levels varied among different cell lines with the lowest in DMBC11 cells and the highest in DMBC28 and DMBC22 cells (Figure 1D). Using the whole-exome sequencing data [24], we have also determined the alterations in genes encoding proteins related to the NF-κB signaling pathway. We have found a number of variants leading to amino acid substitutions in interleukin-1β (IL-1β) (Figure 1E and Table S2). Heterozygous IκB kinase alpha (IKKα^P364S^) and toll-like receptor 4 (TLR4^P202T^) variants were found exclusively in DMBC22 cells, whilst only these cells harbored *NFKBIE* (encoding IκBε) as a wild-type. In addition, a homozygous frameshift variant of nuclear factor-kappa B-inducing kinase (NIK) was found in all cell lines (Figure 1E and Table S2).

### 2.2. 17-Aminogeldanamycin (AG) Reduces Viable Cell Number in Melanoma Cell Lines, which is Associated with Induction of Apoptosis

**Figure 2:**
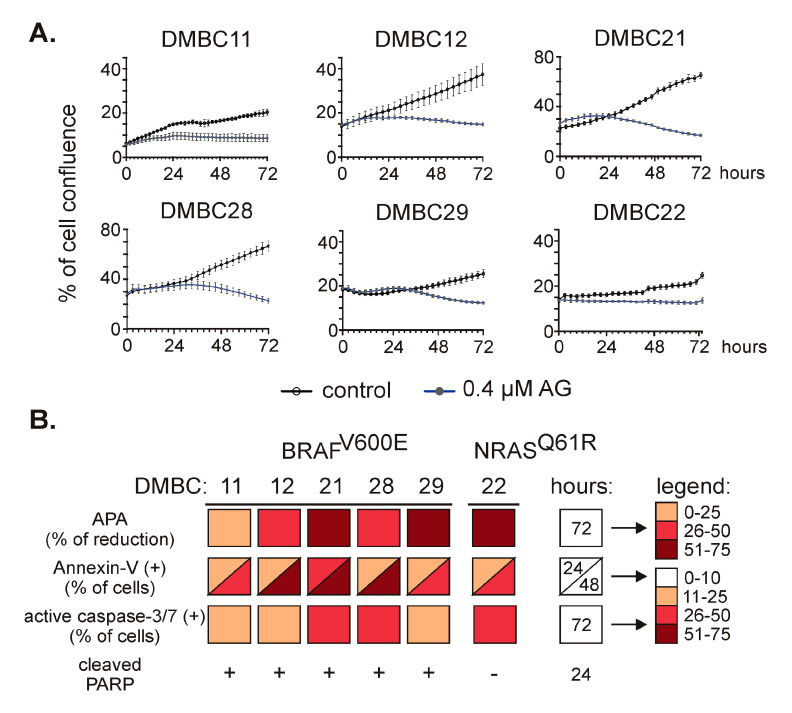
The effect of 17-aminogeldanamycin (AG) on cell viability in BRAF^V600E^ and NRAS^Q61R^ melanoma cell lines. (**A**) Melanoma cells were exposed to AG at 0.4 μM. Cell confluence was assessed over the course of 72 h by time-lapse imaging system IncuCyte ZOOM. Data presented are mean ± SD of a representative experiment performed in duplicate; *n* = 3. (**B**) Cell viability and induction of apoptosis were assessed in melanoma cell cultures exposed to AG at 0.4 μM using acid phosphatase activity (APA) assay, annexin-V/propidium iodide staining followed by flow cytometry, real-time fluorescence microscopy (% of cells with active caspase-3/7), and Western blotting (cleaved PARP). The color-coded summary of the results is shown at indicated time points. The percentages of annexin-V-positive cells and cells with active caspase-3/7 in the controls did not exceed 10%. The original results for DMBC11 cells are shown in Figure S1; for other cell lines, they are published elsewhere [21].

To investigate the effect of 17-aminogeldanamycin (AG), we monitored changes in viable cell numbers using time-lapse imaging. AG at 0.4 µM efficiently reduced cell confluence in all six cell lines (Figure 2A) assigned to either *BRAF* or *NRAS* subtypes, and growth inhibition reached about 50% of control after 72 h. Growth inhibition was additionally confirmed by the assessment of acid phosphatase activity in DMBC11 (Figure S1A) and other cell lines [21]. This was associated with induction of apoptosis as melanoma cell lines exposed to AG at 0.4 µM exhibited (i) a time-dependent increase in the percentages of annexin-V-positive cells, (ii) elevated percentages of cells with active caspase-3/7, and (iii) increased level of cleaved poly-(ADP-ribose) polymerase (PARP). These cellular and molecular effects are collectively shown in Figure 2B.

### 2.3. AG Inhibits p65/NF-κB Activity in Melanoma Cells of BRAF^V600E^ and NRAS^Q61R^ Subtypes

**Figure 3:**
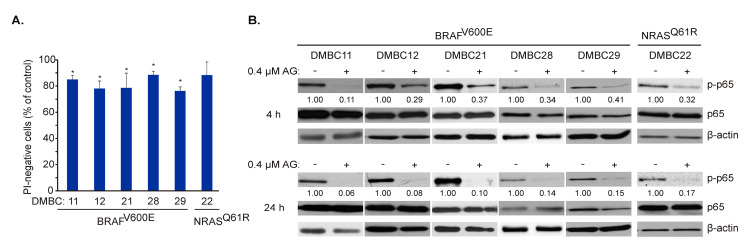
17-Aminogeldanamycin (AG) inhibits the activity of p65/NF-κB in melanoma cell lines of the BRAF^V600E^ or NRAS^Q61R^ subtypes before it substantially affects cell viability. (**A**) Cells were incubated with AG at 0.4 μM for 24 h. Propidium iodide (PI)-negative (viable) cells were assessed by flow cytometry and expressed relative to the control. Data presented are mean ± SD; *n* = 3 (* *p* ≤ 0.05 vs. control). (**B**) Levels of phosphorylated p65 (p-p65) and total p65 were determined by Western blotting after 4 and 24 h. β-actin was used as a loading control. The relative level of p-p65 versus β-actin is shown below the blots.

AG at 0.4 µM did not induce the massive cell death in melanoma cells after 24 h (Figure 2B) that was additionally confirmed in all six cell lines by propidium iodide (PI) staining followed by flow cytometry (Figure 3A). Therefore, we focused on molecular events at time intervals, not exceeding this time point. AG at 0.4 μM substantially diminished constitutive activity of NF-κB already after 4 h, as assessed at the level of phosphorylated p65 (Figure 3B upper panel). This effect was enhanced after an additional 20 h of incubation, indicating a complete attenuation of NF-κB activity in cell lines harboring either BRAF^V600E^ or NRAS^Q61R^ variants (Figure 3B lower panel). Notably, total p65 protein level remained unaltered, even after 24 h of incubation with AG (Figure 3B).

### 2.4. AG Diversely Affects Transcript and Proteins Levels of Different NF-κB-Dependent Genes

**Figure 4:**
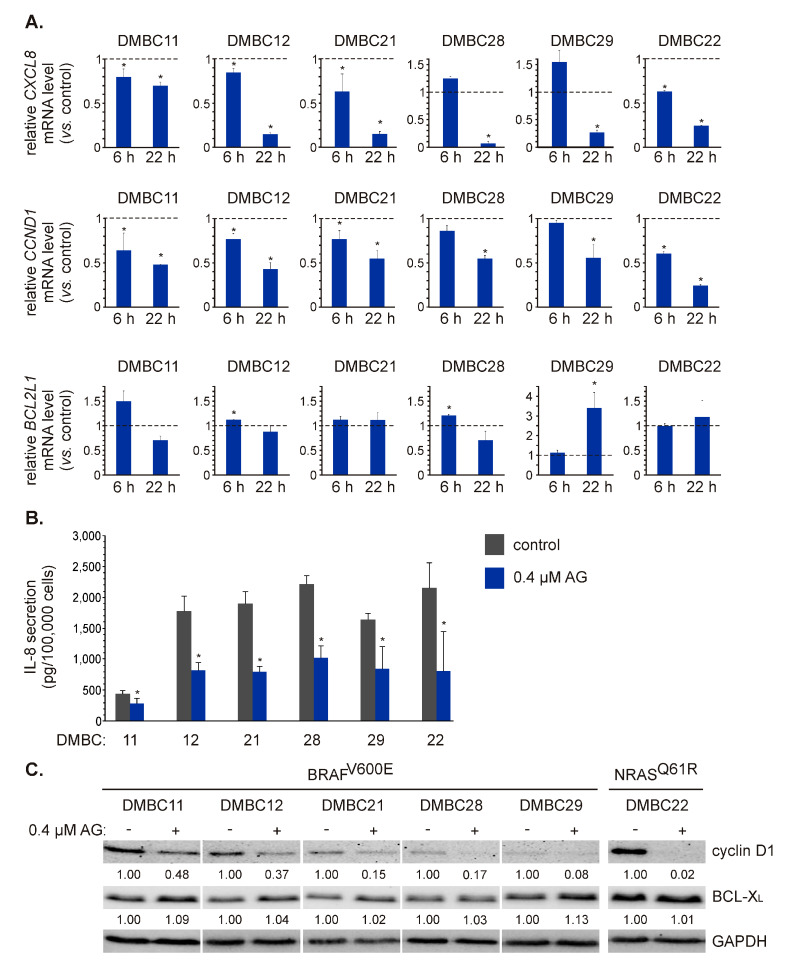
The effect of 17-aminogeldanamycin (AG) on mRNA and protein levels of NF-κB-dependent genes in melanoma cells of the BRAF^V600E^ or NRAS^Q61R^ subtype. (**A**) The transcript levels of *CXCL8* (IL-8), *CCND1* (cyclin D1), and *BCL2L1* (BCL-X_L_) were assessed by qRT-PCR after 6 and 22 h and shown relative to the control. Data presented are mean ± SD; *n* = 3 (* *p* ≤ 0.05 vs. control). (**B**) Samples of the culture medium were collected after 24 h of cell incubation with 0.4 μM AG. The protein levels of IL-8 were determined by enzyme-linked immunosorbent assay (ELISA). Data presented are mean ± SD; *n* = 3 (* *p* ≤ 0.05 vs. control). (**C**) The protein levels of cyclin D1 and BCL-X_L_ were determined by Western blotting after 24 h. GAPDH was used as a loading control. The relative levels of cyclin D1/BCL-X_L_ *versus* GAPDH are shown below the blots as an average of two independent experiments.

To validate AG-induced effects on p65/NF-κB activity, we assessed the expression of three NF-κB-regulated genes involved in different cellular processes: *CXCL8*, *CCND1,* and *BCL2L1*. AG at 0.4 μM significantly decreased the transcript level of *CXCL8* after 22 h in BRAF^V600E^ and NRAS^Q61R^ cell lines (Figure 4A). This effect was, however, much less pronounced in DMBC11 cells compared with other cell lines (Figure 4A) that might be associated with already the lowest initial level of the *CXCL8* transcript in these cells (Figure 1D). Also, the concentration of interleukin-8 (IL-8) protein in the medium of DMBC11 cell culture was the lowest compared with other cell lines (Figure 4B). AG at 0.4 μM significantly reduced the levels of secreted IL-8 in all melanoma cell cultures after 24 h (Figure 4B). AG at this concentration also significantly downregulated *CCND1* expression already after 6 h in the majority of melanoma cell lines (Figure 4A). *CCND1* mRNA level was further reduced to about 50% of control (*p* ≤ 0.05) after 22 h in all melanoma cell lines, or even below 50% of control as in DMBC22 cells (Figure 4A). Accordingly, protein levels of cyclin D1 were substantially reduced in melanoma cell lines exposed to AG (Figure 4C).

AG at 0.4 μM did not substantially affect the transcript level of *BCL2L1* in most cell lines, except for DMBC29 cells in which *BCL2L1* was significantly upregulated in response to AG exposure for 22 h (Figure 4A). At the protein level, however, no substantial changes were induced in any cell line after exposure to AG (Figure 4C). Notably, the original geldanamycin at 0.4 μM did not affect the mRNA levels of *CXCL8*, *CCND1,* and *BCL2L1* in DMBC21 and DMBC29 cells after 22 h (Figure S2), which indicates that observed effects could be induced only by its derivative investigated in this study.

### 2.5. AG Affects Transcript Level and Secretion of VEGF

**Figure 5:**
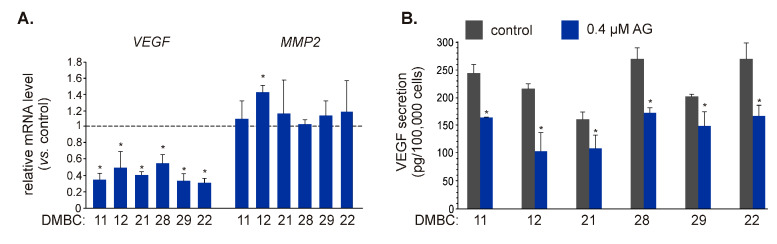
The influence of 17-aminogeldanamycin (AG) on the expression of NF-κB-dependent genes that control angiogenesis and invasion. (**A**) The transcript levels of vascular endothelial growth factor (*VEGF*) and matrix metalloproteinase-2 (*MMP2*) were assessed by qRT-PCR after 22 h and shown relative to the control. Data presented are mean ± SD; *n* = 3 (* *p* ≤ 0.05 vs. control). (**B**) Samples of the culture medium were collected after 24 h of melanoma cell incubation with 0.4 μM AG. The levels of VEGF protein were determined by ELISA. Data presented are mean ± SD; *n* = 3 (* *p* ≤ 0.05 vs. control).

As IL-8 can regulate different cellular programs, including invasion/migration and angiogenesis [33,34], and AG significantly downregulated expression of *CXCL8* and reduced the amount of IL-8 protein secreted by melanoma cells, we aimed to assess the influence of AG on other regulators of these processes. Vascular endothelial growth factor (VEGF) plays an essential role in angiogenesis [35,36], while matrix metalloproteinase-2 (MMP-2) promotes cell invasion and migration by digesting the extracellular matrix [37]. Both *VEGF* [38] and *MMP2* [39,40] have been identified as NF-κB target genes. AG at 0.4 μM significantly reduced transcript level of *VEGF* in BRAF^V600E^ and NRAS^Q61R^ melanoma cells (Figure 5A), and notably, it also reduced (*p* ≤ 0.05) the amount of VEGF protein in the culture medium (Figure 5B). In contrast, the *MMP2* mRNA level was not substantially affected in any cell line exposed to AG for 22 h (Figure 5A).

## 3. Discussion

In the present study, we have demonstrated that 17-aminogeldanamycin inhibits a constitutive p65/NF-κB activity in BRAF^V600E^ and NRAS^Q61R^ melanoma cell lines that is evidenced by (i) attenuation of the level of phosphorylated p65/NF-κB subunit, (ii) decrease of transcript levels of NF-κB-dependent genes, including *CCND1, CXCL8,* and *VEGF*, (iii) diminution of the level of corresponding proteins in melanoma cells, as well as (iv) reduced secretion of IL-8 and VEGF.

The NF-κB signaling pathway is constitutively active in melanoma cells, where it regulates the expression of genes that control proliferation, cell cycle, survival and apoptosis, inflammation, invasion, and angiogenesis [41,42,43,44]. The role of NF-κB is, however, complex and largely context-dependent. It has been demonstrated that NF-κB activity is preferentially maintained in a subpopulation of ATP-binding cassette member B5 (ABCB5)-positive melanoma cells, which exert more metastatic phenotype than ABCB5-deficient cells [45]. The activation of NF-κB has been implicated in acquired resistance of melanoma cells to BRAF^V600^ inhibitors [46,47,48] and occurs at the early stage of adaptation to treatment [49]. In addition, NF-κB is involved in the interferon-γ (IFN-γ)-inducible expression of programmed death-ligand-1 (PD-L1) in melanoma cells [50,51]. In the present study, we focused on a p65 subunit of NF-κB and its phosphorylation at Ser^536^ as a readout of NF-κB activity. Phosphorylation of p65 at this residue promotes a conformational switch that enables nuclear translocation of NF-κB and binding to the promoter regions of NF-κB-inducible genes [52,53,54]. It has been also demonstrated that p65 as a part of p65-p50 heterodimer regulates expression of genes that contribute to the metastatic phenotype of melanoma cells that is a consequence of the low level of KIP1 ubiquitination-promoting complex protein 1 (KPC1) associated with limited availability of p50-p50 homodimer involved in suppression of NF-κB-dependent cell proliferation [55]. Patient-derived melanoma cell lines used in the present study exhibited a constitutive activity of NF-κB that is in agreement with our previous studies reporting substantial levels of phosphorylated p65 in cells harboring mutations in either *BRAF* or *RAS* [22,23], and indicating that NF-κB activity is independent of extracellular growth factor stimuli [56]. We have extended characteristics of investigated cell lines by validating activity of the NF-κB signaling pathway at the level of (i) genetic alterations in genes encoding proteins related to the NF-κB pathway based on the KEGG PATHWAY database, and (ii) assessment of expression of 84 NF-κB-inducible genes. We have found a large diversity of transcript levels between three representative melanoma cell lines, and this was also observed when transcript levels of three selected genes were assessed in six cell lines of either the *BRAF* or *NRAS* subtype. This diversity, particularly exemplified by mRNA levels of *CXCL8*, can result from the contribution of other transcriptional regulators and/or genetic alterations in these cells. In melanoma, *CXCL8* expression can be regulated by nuclear factor of activated T-cells 1 (NFAT1) [57], signal transducer and activator of transcription 3 (STAT3) [58] and WNT signaling pathway [59]. In addition, expression of *CXCL8* can be stimulated by various cytokines that activate NF-κB, including IL-1, interleukin-6 (IL-6), and tumor necrosis factor alpha (TNFα) [33]. In this respect, genetic alterations diversely distributed among melanoma cell lines might determine different levels of *CXCL8* transcript as DMBC12, DMBC21, DMBC29, and DMBC22 cell lines harbored several point mutations in *IL1B*. IL-1β, a product of *IL1B* expression, is secreted by melanoma and stroma cells and induces NF-κB following IL-1R activation in an autocrine or paracrine manner [60,61,62]. In addition, we have also demonstrated that the expression of *IL1B* substantially varied among melanoma cell lines. We have found that the mRNA level of *IL1B* was 4.1-fold and 7-fold higher in DMBC12 and DMBC21 cell lines, respectively, when compared to its level in the DMBC11 cell line (Figure 1C and Table 1). The lowest level of *CXCL8* mRNA in DMBC11 cells, and consistently the lowest amount of IL-8 secreted by these cells, might be associated with low expression of *IL1B* in these cells. Other genetic alterations identified in our study can also putatively affect the activity of NF-κB signaling. Notably, a frameshift variant of NIK was harbored in all cell lines, suggesting that it might contribute to the constitutive activity of NF-κB as NIK has been identified as a regulator of NF-κB activation via both the MAPK pathway and canonical IKK/IκB cascade in melanoma [63]. More recently, it has been shown that NIK also modulates the non-canonical NF-κB signaling pathway and β-catenin-dependent transcription of genes encoding pro-survival proteins [64]. In addition, IKKα^P364S^ and TLR4^P202T^ variants were harbored exclusively in DMBC22 cells that exhibited the highest level of *CXCL8*, *CCND1,* and *BCL2L1* mRNA. It has also been recently reported that G34E substitution in IκBε promoted NF-κB-dependent expression of *CD83* that was associated with patient responsiveness to immunotherapy [65]. In our study, this variant was harbored in DMBC11 and DMBC12 cells, but the expression of *CD83* was the most substantially upregulated in DMBC21 (Figure 1C and Table 1) that harbored another variant of IκBε (S33F), also predicted as damaging according to the PolyPhen-2 software. The clinical utility of this variant and the functional roles of other genetic alterations reported in the present study, however, need to be determined.

The activity of NF-κB signaling can be regulated by HSP90, which is involved in biogenesis and activation of IKKs that liberate the NF-κB transcription factor from an inhibitory complex with IκB proteins [66]. Accordingly, inhibition of NF-κB activity has already been demonstrated for other HSP90 inhibitors [67,68], and we are the first to report that 17-aminogeldanamycin potently attenuates the level of phospho-p65/NF-κB in melanoma cells. We have also demonstrated that 17-aminogeldanamycin does not affect total levels of p65 suggesting the influence of this compound on upstream regulators of the p65 activation. There are a few potential mechanisms. It has been reported that Ser^536^ phosphorylation is independent of IκB degradation [69], and such a mechanism of NF-κB inhibition by geldanamycin has been demonstrated [67]. In addition, phosphorylation of p65 at Ser^536^ is managed in the cytoplasm by a plethora of kinases such as TANK-binding kinase 1 (TBK1), ribosomal kinase 1 (RSK1) and AKT1, in addition to IKK [70,71,72]. Moreover, cyclin-dependent kinase 6 (CDK6) phosphorylates p65 at Ser^536^ in the nucleus and this phosphorylation of p65 favors the expression of inflammatory genes [73,74]. All mentioned kinases that phosphorylate p65 at Ser^536^ have already been identified as HSP90 client proteins (www.picard.ch); thus, they may play a role in the phosphorylation of p65 at Ser^536^ in melanoma. To validate the consequences of NF-κB inhibition by 17-aminogeldanamycin, we have assessed the expression of three selected genes encoding proteins that regulate different cellular processes. The expression of *CXCL8* and *CCND1* was significantly diminished in all melanoma cell lines, irrespective of their basal mRNA and protein levels that confirmed the inhibitory effect of 17-aminogeldanamycin on NF-κB activity. Surprisingly, the level of *BCL2L1* transcript remained mostly unaltered or was even significantly upregulated, as shown in DMBC29 cells. A consistent lack of a significant decrease in the BCL-X_L_ transcript level in melanoma cells exposed to 17-aminogeldanamycin might be explained by different NF-κB dimer compositions required for the activation of *BCL2L1* expression. This is in line with a study showing that the dimerization of p65 with p50 did not affect BCL-X_L_ level while coupling p65 (RelA) with c-Rel upregulated *BCL2L1* expression [75]. In addition, *BCL2L1* is regulated by the paired box 3 (PAX3) transcription factor [76]. Expression of both genes has been shown to increase in melanoma [77], while siRNA-mediated PAX3 downmodulation significantly decreased the expression of *BCL2L1* in several melanoma cell lines [78]. It has also been demonstrated that the BCL-X_L_ level was positively correlated with the level of active STAT3 in melanoma [79]. As the expression of *BCL2L1* was markedly increased only in DMBC29 cells exposed to 17-aminogeldanamycin, these putative mechanisms might be, however, diversely involved in the regulation of BCL-X_L_ level in different melanoma cell lines. In addition, we have recently reported that an S412C variant of enhancer of zeste homolog 2 (EZH2) is exclusively harbored in DMBC29 cells [24]. EZH2 is a member of polycomb repressive complex 2 (PRC2) that methylates histone H3 at lysine 27, thus acts as a gene repressor [80]. Overexpression and gain-of-function mutations of *EZH2* are typical for many cancers, including melanoma [81,82,83]. Moreover, EZH2 may act in a histone methyltransferase-independent way as a partner of p65/NF-κB to increase the level of NF-κB-inducible transcripts [84]. Notably, wild-type EZH2 is also a client protein for HSP90, and disruption of EZH2–HSP90 interaction destabilized EZH2 and led to its degradation [85,86]. It has also been shown that disruption of processes controlled by a wild-type EZH2 resulted in the reduction of BCL-X_L_ level [87], suggesting a role of EZH2 in the regulation of *BCL2L1* expression. It should also be considered that BCL-X_L_ is substantially more stable [30,88] than either IL-8 [34] or cyclin D1 [89], suggesting that changes in mRNA and protein levels assessed after 22–24 h might also reflect differences in the half-lives of transcripts and proteins.

**Figure 6:**
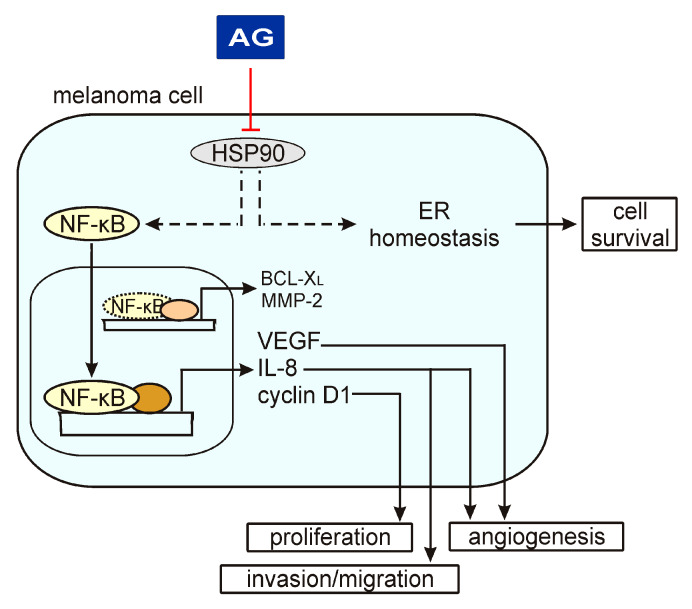
A schematic model of AG activity in melanoma cells based on the results shown in this study and published previously [21]. A heat shock protein 90 (HSP90) has been shown to control the p65/NF-κB signaling pathway and endoplasmic reticulum (ER) homeostasis, among others. AG inhibits HSP90 activity that is associated with a reduction of phospho-p65 levels and expression of NF-κB target genes, including *VEGF*, *CXCL8,* and *CCND1*. In this way, AG can affect different cellular programs. In turn, AG does not change the levels of BCL-X_L_ and MMP-2, suggesting that regulators other than NF-κB play a dominant role in the activation of their expression.

17-Aminogeldanamycin-induced changes in the expression of the NF-κB-inducible genes assessed in the present study might have substantial consequences for the execution of different cellular programs in melanoma (Figure 6). Downregulation of *CCND1* mechanistically links the cytostatic activity of this HSP90 inhibitor evidenced by a decreased number of viable cells shown in this study and elsewhere [20,21]. This, however, can also be associated with the induction of cell death. Our study suggests that apoptosis induced in melanoma cells by 17-aminogeldanamycin is not accompanied by changes in the BCL-X_L_ level. In turn, we have recently shown that 17-aminogeldanamycin induces apoptotic cell death that is associated with the attenuation of cytoprotective inositol-requiring enzyme 1 alpha (IRE-1α)/spliced X-box binding protein 1 (XBP1s) axis in response to endoplasmic reticulum (ER) stress triggered by HSP90 inhibition [21]. In the present study, we have also demonstrated that 17-aminogeldanamycin reduces transcript and protein levels of VEGF, in addition to the downregulation of *CXCL8* expression, while it does not affect the expression of *MMP2*. This suggests that HSP90 inhibition by this compound might predominantly affect molecular pathways that promote angiogenesis.

It has been recently demonstrated that IL-8 mediates invasion of melanoma cells and angiogenesis in a zebrafish model of melanoma, while downregulation of *CXCL8* expression was sufficient to counteract the invasive capability of BCL-X_L_^high^ melanoma cells [90]. In this respect, 17-aminogeldanamycin-mediated reduction of extracellular levels of IL-8 may be sufficient to hamper the effects of BCL-X_L_ activity, even if *BCL2L1* expression is not affected by this compound. Additionally, cooperation between VEGF and IL-8 has been evidenced to expand tumor vasculature [91,92], and IL-8 can promote the expression of *VEGF* in an NF-κB-dependent manner in endothelial cells [93]. As a consequence, inhibition of VEGF secretion was shown to be associated with reduced branching and diminished formation of capillary vessels within tumors [94]. In melanoma patients, serum levels of VEGF correlated with the stage of the disease [95]. The pro-angiogenic role of VEGF, including VEGF-dependent formation of vessel-like structures by cancer cells known as vasculogenic mimicry (VM), has been extensively evidenced in melanoma [96,97,98,99,100,101,102]. VM has been shown to modulate the sensitivity of melanoma cells to drugs [103], but also demonstrated as an adaptive response to VEGF depletion in melanoma [104]. Most recently, it has been shown that elevated levels of vascular endothelial growth factor receptor-1 (VEGFR-1) rendered resistance to vemurafenib in melanoma cells, which additionally secreted more VEGF than their drug-sensitive counterparts [105]. In addition, circulating VEGF has been indicated as a predictive biomarker for a response of melanoma patients to BRAF/MEK inhibitors, and for monitoring the onset of drug resistance [106]. Notably, a mechanistic contribution of NF-κB to VM [107,108] suggests that compounds modulating the activity of this transcription factor may affect VM/angiogenesis in melanoma. Although further research is necessary to more extensively delineate the cellular effects of 17-aminogeldanamycin, our study provides molecular evidence that this HSP90 inhibitor can be used for the simultaneous diminution of IL-8 and VEGF levels in an extracellular milieu to reach substantial anti-melanoma effects.

## 4. Materials and Methods

### 4.1. Cell Culture

Melanoma cell lines were derived from surgical specimens, as described previously [109]. The study was approved by the Ethical Commission of Medical University of Lodz (identification code: RNN/84/09/KE). Informed consent was obtained from all patients. Cell lines were named DMBC (Department of Molecular Biology of Cancer) and maintained in the stem cell medium (SCM) consisting of DMEM/F12 medium (Gibco, Paisley, UK), B-27 supplement (Gibco), 1 ng/mL heparin, 10 μg/mL insulin, 10 ng/mL bFGF, 20 ng/mL EGF (BD Biosciences, San Jose, CA, USA), 100 μg/mL streptomycin, and 100 IU/mL penicillin. Cell lines were assigned to the *BRAF* (DMBC11, DMBC12, DMBC21, DMBC28, and DMBC29) or *NRAS* (DMBC22) subtype [24]. Specifically, a homozygous BRAF^V600E^ variant was harbored in DMBC11 and DMBC12 cells, whereas a heterozygous BRAF^V600E^ was found in DMBC21, DMBC28, and DMBC29 cells. A homozygous Q61R substitution in NRAS was harbored in DMBC22 cells [24]. For experiments, melanoma cells were exposed to 0.4 μM 17-aminogeldanamycin (AG) to monitor changes in cell confluence and viability up to 72 h, or collected for RNA extraction (after 6 and 22 h), protein lysate preparation (after 4 and 24 h) and flow cytometry analysis (after 24 h). DMBC21 and DMBC29 cells were also exposed to 0.4 μM geldanamycin for 22 h, followed by isolation of RNA and analysis of gene expression.

### 4.2. Compounds

17-Aminogeldanamycin was purchased from BOC Sciences (Shirley, NY, USA), and geldanamycin was purchased from Sigma-Aldrich (St Louis, MO, USA).

### 4.3. Time-Lapse Microscopy

Melanoma cells were grown in 96-well plates (8 × 10^3^ cells/well) and exposed to AG at 0.4 µM for 72 h. Cell confluence was monitored by using a time-lapse microscope system IncuCyte ZOOM (IncuCyte, Essen Bioscience, Essen, Germany). Data were analyzed using the IncuCyte Zoom original software. For apoptosis assay, IncuCyte Caspase-3/7 Apoptosis Assay Reagent at 4 μM was additionally added into wells. Then, % of cells with active caspase-3/7 was expressed as the percentage of the confluence of apoptotic cells divided by the percentage of the confluence of all cells.

### 4.4. Acid Phosphatase Activity Assay

To assess cell viability, the activity of acid phosphatase was measured colorimetrically, as described previously [109].

### 4.5. Flow Cytometry

Cells were incubated with 17-aminogeldanamycin for 24 h, then collected, trypsinized, and stained with propidium iodide (Sigma-Aldrich) for 5 min in the dark. To determine the percentages of annexin-V-positive cells, melanoma cells were incubated with the staining solution containing Annexin-V and propidium iodide (BD Biosciences) for 15 min. Flow cytometric data were acquired with FACSVerse (BD Biosciences) and analyzed using the BD FACSuite software.

### 4.6. Cell Lysate Preparation and Western Blotting

Melanoma cells were subjected to 30-min lysis at 4 °C by using RIPA buffer (50 mmol/L Tris-HCl, pH 8.0, 150 mmol/L NaCl, 1% Triton X-100, 0.5% sodium deoxycholate, 0.1% SDS), containing freshly added protease and phosphatase inhibitors (Sigma-Aldrich). Then, cell lysates were centrifuged (14,000 rpm, 15 min, 4 °C), and protein concentration was determined by using Bradford assay (BioRad, Hercules, California, USA). Cell lysates were diluted in 2× Laemmli sample buffer (125 mmol/L Tris-HCl, pH 6.8, 0.004% bromophenol blue, 20% glycerol, 4% SDS, and 10% β-mercaptoethanol). Samples of 15 µg were loaded onto 7% SDS-polyacrylamide gel, and electrophoresis was run at a constant voltage of 25V/cm. Next, the proteins were transferred to an Immobilon-P PVDF membrane (Millipore, Billerica, Massachusetts, USA; 100 V, 1 h, 4 °C) using BioRad transfer equipment. The membrane was stained with 0.1% Ponceau S to confirm effective protein transfer. Then, the membrane was incubated in a blocking solution: 5% non-fat milk in PBS-Tween 0.05% or 5% phospho-BLOCKER (Cell Biolabs, San Diego, CA, USA) in PBS-Tween 0.05% for 1 h. Primary antibodies detecting phosphorylated p65 (p-p65 Ser536), total p65, cyclin D1, BCL-X_L_ (Cell Signaling Technology, Danvers, MA, USA), GAPDH, PARP (Santa Cruz Biotechnology, Santa Cruz, California, USA), and β-actin (Sigma-Aldrich) were used followed by binding of the secondary HRP-conjugated anti-mouse or anti-rabbit antibodies (Santa Cruz Biotechnology). The proteins were visualized on a medical X-ray film (Foton-Bis, Bydgoszcz, Poland) or by using a ChemiDoc Imaging System (Biorad). Pierce ECL Western Blotting Substrate (Pierce, Rockford, Illinois, USA) was used as a chemiluminescence reagent. The quantification of the Western blotting data was performed by using ImageJ software and is shown relative to GAPDH or β-actin.

### 4.7. RNA Isolation, Synthesis of cDNA and Quantitative Real-Time PCR (qRT-PCR)

Total RNA was extracted using a Total RNA Isolation Kit with a mini-column system (A&A Biotechnology, Gdynia, Poland). RNA concentration and purity were determined by using NanoQuant Plate Infinite M200 PRO reader (Tecan Austria GmbH, Grodig, Austria) and the corresponding software. Total RNA (1 μg) was transcribed into cDNA using 300 ng of random primers and SuperScript II Reverse Transcriptase (Invitrogen Life Technologies, Carlsbad, CA, USA). The evaluation of the mRNA level for selected genes was performed by quantitative real-time polymerase chain reaction (qRT-PCR) by using a Rotor-Gene 3000 Real-Time DNA analysis system (Corbett Research, Morklake, Australia). cDNA was amplified with KAPA SYBR FAST qPCR Kit Universal 2× qPCR Master Mix (Kapa Biosystems, Cape Town, South Africa). We used 200 nM of each primer and 25 ng cDNA template per reaction. The annealing temperature for all transcripts was 56 °C. Primer sequences for *RPS17*, *CXCL8*, *CCND1, VEGF, MMP2,* and *BCL2L1* are published elsewhere [27,30,110].

### 4.8. Enzyme-Linked Immunosorbent Assay (ELISA)

ELISA kits Quantikine High Sensitivity Human CXCL8/IL-8 and Human VEGF Immunoassay (R&D Systems, Minneapolis, MN, USA) were used to determine IL-8 and VEGF secretion by melanoma cells to the culture medium after 24 h. The procedures were performed according to the manufacturer’s instructions. For the quantification of IL-8, medium samples were 40× diluted. The optical density of each well was determined using a microplate reader Infinite M200Pro (Tecan) set to 490 nm with wavelength correction at 650 nm (for IL-8 assay) or 450 nm with wavelength correction at 540 nm (for VEGF assay). Concentrations of IL-8 and VEGF in the medium samples were calculated using a four-parameter logistic (4-PL) curve fit and expressed as picograms (pg) per 100,000 cells.

### 4.9. RT^2^ Profiler PCR Array

RNA was extracted, and cDNA was synthesized as described in Section 4.7. A NF-κB Signaling Targets RT^2^ Profiler PCR array was performed according to the manufacturer protocol (Qiagen, Hilden, Germany). In brief, cDNA solution (25 ng/µL) was dissolved in double-distilled nuclease-free water, and mixed with Master Mix. The solution was immediately added into the wells (5 ng of cDNA per well) of the 96-well plate containing specific primer pairs (Table S1). The plate was covered with adhesive optical film, and raw data were acquired by using an ABI 7900HT Fast Real-Time PCR System (Applied Biosystems). PCR array data analysis platform provided by Qiagen was used to normalize and visualize data. Raw data were normalized to a reference gene, *B2M*. Heatmaps were prepared based on the log_2_-transformed fold-change values to visualize differentially expressed genes in DMBC12 and DMBC21 cells relative to transcript levels in DMBC11 cells. Scatter plots were obtained by log_10_ normalization of gene expression.

### 4.10. Whole-Exome Sequencing

Whole-exome sequencing was described previously [24]. In brief, DNA was extracted from melanoma cells using a DNeasy Blood and Tissue kit (Qiagen), and DNA concentration and purity were determined using a NanoQuant Plate Infinite M200 PRO reader (Tecan Austria GmbH). DNA sequencing was performed by ABM Good (Richmond, Canada). DNA samples were subjected to Nextera tagmentation, followed by index PCR by using Nextera Rapid Capture Exome (Illumina). The amplified DNA was assessed by Agilent 2100 Bioanalyzer (Agilent Technologies) to determine size distribution, and quantification of the library concentration was performed using KAPA SYBR FAST qPCR kit (Kapa Biosystems). The cluster generation and two-channel sequencing were performed using NextSeq 500 (Illumina). Data were mapped to the reference genome (version GRCh37/hg19) using the BWA package. Indel realignment and base recalibration were performed using GATK. VCF files were generated to identify single nucleotide variants (SNVs) and short insertions or deletions (indels). Raw data are freely available at ArrayExpress under the accession number E-MTAB-6978 and European Nucleotide Archive (ENA) under the number ERP109743. The functional consequences of amino acid substitutions were predicted in silico using the PolyPhen-2 software available online (genetics.bwh.harvard.edu/pph2/index.shtml). The PolyPhen-2-based predictions were classified as benign (scores 0.000–0.449), possibly damaging (scores 0.450–0.959) or probably damaging (scores 0.960–1.000).

### 4.11. Statistical Analysis

Graphs are presented as mean ± SD. The unpaired *t*-test was used to compare two samples. The differences were considered significant if *p* ≤ 0.05.

## 5. Conclusions

This is the first study that investigates the activity of 17-aminogeldanamycin on the p65/NF-κB program in melanoma cell lines of genetically different subtypes, *BRAF* and *RAS*. Our report indicates that 17-aminogeldanamycin can counteract the effects of NF-κB activity mediated by NF-κB-dependent genes, encoding proteins involved in cell proliferation, invasion, and angiogenesis. Thus, 17-aminogeldanamycin might be considered as a single agent or an adjuvant therapy in melanoma.

## Figures and Tables

**Table 1:** Genes over- and underexpressed in DMBC12 and DMBC21 cell lines compared to DMBC11 cells. The cut-off fold change was set as 2.

Genes Overexpressed in DMBC12 vs. DMBC11		Genes Underexpressed in DMBC12 vs. DMBC11	
Gene Symbol	Fold Regulation	Gene Symbol	Fold Regulation
*CXCL8*	42.64	*ICAM1*	−5.60
*CXCL1*	25.41	*TNFSF10*	−4.50
*CXCL2*	13.27	*IL6*	−3.74
*BIRC3*	12.79	*IL4*	−3.68
*CCL2*	5.28	*IL12B*	−3.02
*BCL2A1*	4.88	*NQO1*	−3.00
*IL1A*	4.65	*CD69*	−3.11
*EGFR*	4.38	*IL2RA*	−2.52
*IL1B*	4.09	*CCL11*	−2.38
*F8*	2.53	*PLAU*	−2.38
*NFKBIA*	2.23	*FASLG*	−2.27
*NFKB1*	2.10	*IL1RN*	−2.17
		*STAT3*	−2.05
**Genes Overexpressed in DMBC21 vs. DMBC11**		**Genes Underexpressed in DMBC21 vs. DMBC11**	
**Gene Symbol**	**Fold Regulation**	**Gene Symbol**	**Fold Regulation**
*BCL2A1*	70.48	*IL1A*	−43.81
*CCL2*	64.05	*TNFRSF1B*	−15.27
*CXCL1*	46.70	*CCL22*	−10.14
*CXCL2*	26.45	*CCR5*	−7.35
*F8*	22.12	*PLAU*	−6.88
*C3*	19.83	*CD80*	−6.39
*AGT*	15.05	*PTGS2*	−5.34
*CXCL8*	13.33	*IL12B*	−4.77
*IL1B*	7.03	*IL2RA*	−4.72
*ICAM1*	6.19	*FASLG*	−4.69
*NR4A2*	5.18	*CSF3*	−4.60
*ADM*	4.43	*MAP2K6*	−4.25
*IL15*	3.39	*SNAP25*	−4.24
*BCL2L1*	2.61	*EGFR*	−4.12
*SOD2*	2.57	*BIRC3*	−3.68
*CD83*	2.56	*NFKB2*	−3.48
*CDKN1A*	2.44	*CCL11*	−3.34
*STAT1*	2.35	*SELE*	−3.32
		*LTA*	−3.27
		*CSF1*	−3.09
		*GADD45B*	−3.02
		*CSF2*	−2.78
		*IL1RN*	−2.73
		*F3*	−2.54
		*IFNB1*	−2.53
		*ALDH3A2*	−2.33
		*SELP*	−2.21
		*INS*	−2.17
		*CXCL9*	−2.16
		*BIRC2*	−2.11

## Supplementary Materials

Supplementary Materials can be found at https://www.mdpi.com/1422-0067/21/11/3749/s1. Figure S1: Effect of 17-aminogeldanamycin (AG) at 0.4 μM on the viability of DMBC11 cells; Figure S2: Effect of 0.4 μM geldanamycin on mRNA levels of NF-κB-dependent genes, *CXCL8* (IL-8), *CCND1* (cyclin D1), and *BCL2L1* (BCL-XL) in melanoma cells; Table S1: PCR array layout corresponding to Figure 1B; Table S2: Mutation status of genes associated with the NF-κB signaling pathway.

## Abbreviations

ABCB5 AG BCL2L1	ATP-binding cassette member B5 17-aminogeldanamycin BCL-2-like 1
BRAF	B-Raf proto-oncogene serine/threonine kinase
CCND1 CDK6	cyclin D1 cyclin-dependent kinase 6
CXCL8 DMBC ERK1/2 EZH2	C-X-C motif chemokine ligand 8 Department of Molecular Biology of Cancer extracellular signal-regulated kinase 1/2 enhancer of zeste homolog 2
HSP90 IFN-γ IKK IL-1β IL-6 IL-8 MMP-2	heat shock protein 90 interferon-gamma IκB kinase interleukin-1β interleukin-6 interleukin-8 matrix metalloproteinase-2
NF-κB NFAT NIK NRAS PAX3 PD-L1 RSK1 STAT3 TBK1 TNFα VEGF	nuclear factor-kappa B nuclear factor of activated T-cells nuclear factor-kappa B-inducing kinase neuroblastoma RAS viral oncogene homolog paired box 3 programmed death-ligand 1 ribosomal kinase 1 signal transducer and activator of transcription 3 TANK-binding kinase 1 tumor necrosis factor alpha vascular endothelial growth factor
