# Supplementary material for: 17-Aminogeldanamycin Inhibits Constitutive Nuclear Factor-Kappa B (NF-κB) Activity in Patient-Derived Melanoma Cell Lines
Source: Int J Mol Sci. 2020 May 26;21(11):3749. doi: 10.3390/ijms21113749 (PMC7312877; doi:10.3390/ijms21113749)
Supplement: Supplementary file 1 [file ijms-21-03749-s001.pdf]

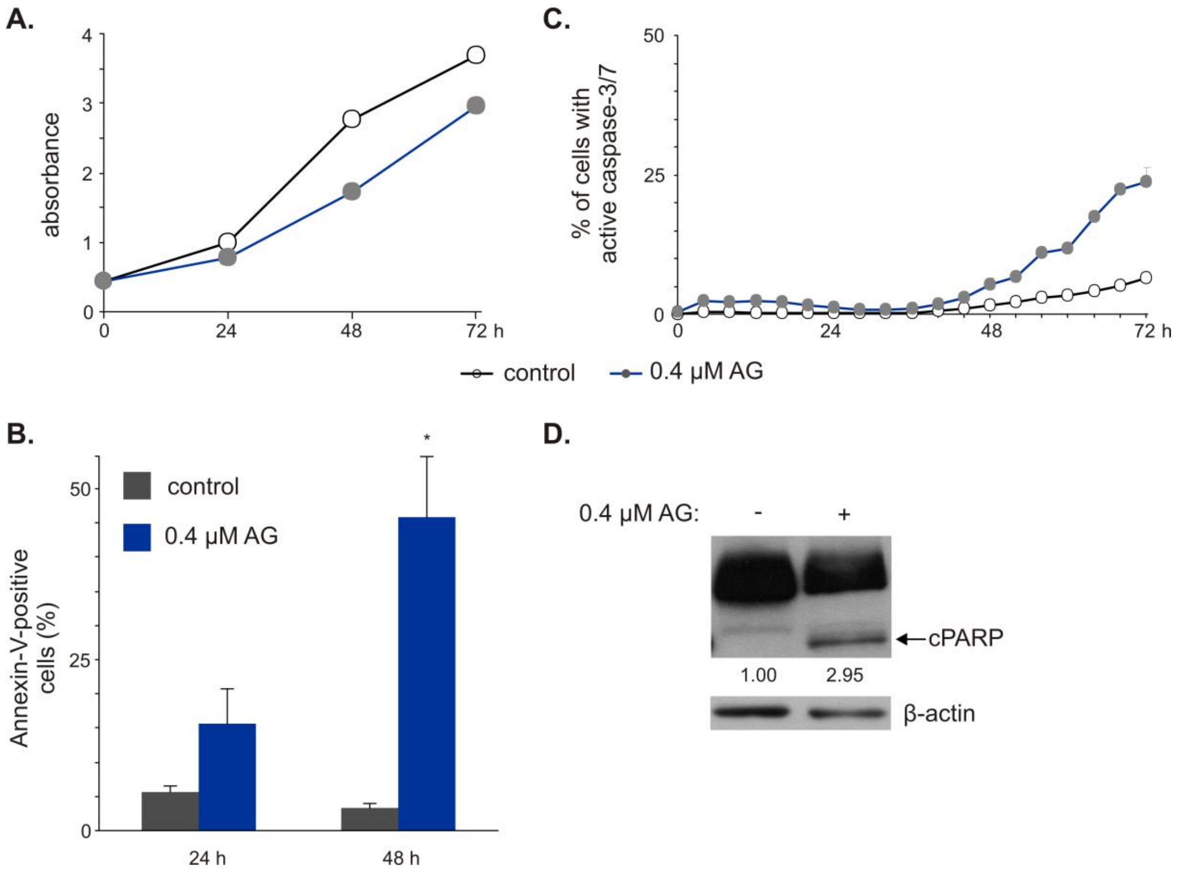

Figure S1. Effect of 17-aminogeldanamycin (AG) at 0.4 µM on viability of DMBC11 cells. **A.** DMBC11 melanoma cells were exposed to AG at 0.4 µM. Acid phosphatase activity (APA) was determined colorimetrically at indicated time points. Data presented are mean ± SD of a representative experiment performed in triplicate. **B.** The percentages of Annexin-V-positive cells were determined by flow cytometry after 24 and 48 hours. n=3 (*$p \leq 0.05$ vs. control). **C.** Activation of caspase-3/7 was assessed over the course of 72 hours by time-lapse imaging system IncuCyte ZOOM. Data presented are mean ± SD of a representative experiment performed in duplicate. **D.** Level of cleaved PARP (cPARP) was determined by Western Blotting after 24 hours. β-actin was used as a loading control. The relative level of cPARP *versus* β-actin is shown below the blot.

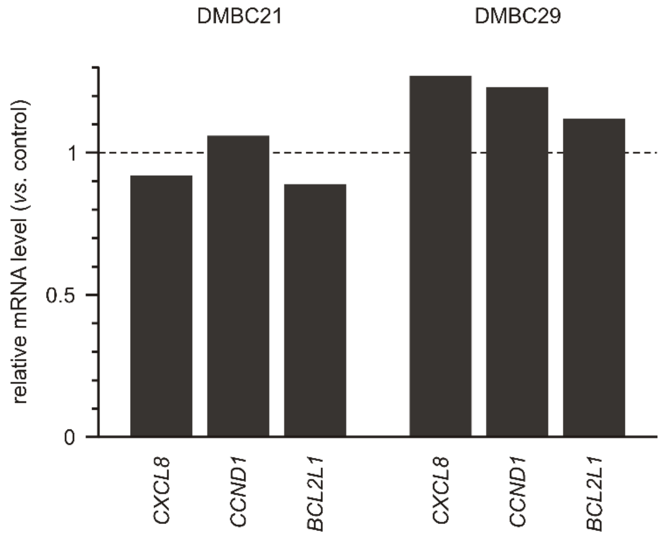

Figure S2. Effect of 0.4 μM geldanamycin on mRNA levels of NF-κB-dependent genes, *CXCL8* (IL-8), *CCND1* (cyclin D1) and *BCL2L1* (BCL-X_L) in melanoma cells. The transcript levels were assessed by qRT-PCR after 22 h, and shown relative to the control.

Table S1. PCR array layout corresponding to Figure 1B.

	**1**	**2**	**3**	**4**	**5**	**6**	**7**	**8**	**9**	**10**	**11**	**12**
**A**	*ADM*	*AGT*	*AKT1*	*ALDH3A2*	*BCL2A1*	*BCL2L1*	*BIRC2*	*BIRC3*	*C3*	*CCL11*	*CCL2*	*CCL22*
**B**	*CCL5*	*CCND1*	*CCR5*	*CD40*	*CD69*	*CD80*	*CD83*	*CDKN1A*	*CFB*	*CSF1*	*CSF2*	*CSF2RB*
**C**	*CSF3*	*CXCL1*	*CXCL10*	*CXCL2*	*CXCL9*	*EGFR*	*EGR2*	*F3*	*F8*	*FAS*	*FASLG*	*GADD458*
**D**	*ICAM1*	*IFNB1*	*IFNG*	*IL12B*	*IL15*	*IL1A*	*IL1B*	*IL1R2*	*IL1RN*	*IL2*	*IL2RA*	*IL4*
**E**	*IL6*	*CXCL8*	*INS*	*IRF1*	*LTA*	*LTB*	*MAP2K6*	*MMP9*	*MYC*	*MYD88*	*NCOA3*	*NFKB1*
**F**	*NFKB2*	*NFKBIA*	*NQO1*	*NR4A2*	*PDGFB*	*PLAU*	*PTGS2*	*REL*	*RELA*	*RELB*	*SELE*	*SELP*
**G**	*SNAP25*	*SOD2*	*STAT1*	*STAT3*	*STAT5B*	*TNF*	*TNFRSF1B*	*TNFSF10*	*TP53*	*TRAF2*	*VCAM1*	*XIAP*

Table S2. Mutation status of genes associated with NF-κB signaling pathway. Only non-synonymous mutations and indels are included. Mutations are marked as homozygous (+/+) or heterozygous (+/-). Nonsense mutation is marked as X. Prediction of functional effects of amino acid substitutions were assessed by using Polyphen-2 software. Polyphen-2 predictions were classified based on the Polyphen-2 scores as benign (scores 0.000-0.449), possibly damaging (scores 0.450-0.959) and probably damaging (scores 0.960-1.000). Names of proteins are given in the brackets if they differ from gene names.

gene	DMBC11	DMBC12	DMBC21	DMBC28	DMBC29	DMBC22
BCL10						
BIRC2 (cIAP1)						
BIRC3 (cIAP2)						
BLNK						
BTK						
CARD11 (CARMA1)						
CD14						
CD40						
CD40LG						
CHUK (IKKα)			V268I +/- benign 0.000	V268I +/- benign 0.000	V268I +/- benign 0.000	V268I +/+ benign 0.000 P364S +/- probably damaging 1.000
CSNK2A1 (CK2)						
CSNK2A2 (CK2)			N63T +/- benign 0.029			
CSNK2A3 (CK2)						
CSNK2B (CK2)						
DDX58 (RIG-1)	R7C +/+ probably damaging 0.997	R7C +/+ probably damaging 0.997 N495K +/- benign 0.025			Q498K +/- benign 0.078	
ERC1 (ELKS)	T1032A +/- benign 0.000	T1032A +/- benign 0.000				
IKBKB (IKKβ)						

IKBKG (NEMO)						
IL1B		Y206N +/-<br>probably damaging 0.999<br>P203H +/-<br>probably damaging 1.000	Y206N +/-<br>probably damaging 0.999		Y206N +/-<br>probably damaging 0.999<br>P203H +/-<br>probably damaging 1.000<br>M211I +/-<br>probably damaging 1.000<br>K209N +/-<br>possibly damaging 0.454<br>K208N +/-<br>probably damaging 0.999	P203H +/-<br>probably damaging 1.000<br>K209N +/-<br>possibly damaging 0.454
IL1R1						
IL1R2						
IRAK1	F196S +/+<br>benign 0.002<br>S532L +/+<br>benign 0.000	F196S +/+<br>benign 0.002<br>S532L +/+<br>benign 0.000	F196S +/-<br>benign 0.002<br>S532L +/+<br>benign 0.000	F196S +/+<br>benign 0.002<br>S532L +/+<br>benign 0.000	F196S +/+<br>benign 0.002<br>S532L +/+<br>benign 0.000	F196S +/+<br>benign 0.002
IRAK4						
LAT						
LBP						
LCK						
LTA						
LTB						
LTBR						
LY96 (MD-2)	R56G +/+<br>benign 0.000	R56G +/+<br>benign 0.000	R56G +/+<br>benign 0.000	R56G +/+<br>benign 0.000	R56G +/+<br>benign 0.000	R56G +/+<br>benign 0.000
LYN						
MALT1						
MAP3K7 (TAK1)						
MAP3K14 (NIK)	R219P/X<br>frameshift variant +/+<br>(insertion)	R219P/X<br>frameshift variant +/+<br>(insertion)	R219P/X<br>frameshift variant +/+<br>(insertion)	R219P/X<br>frameshift variant +/+<br>(insertion)	R219P/X<br>frameshift variant +/+<br>(insertion)	R219P/X<br>frameshift variant +/+<br>(insertion)
MYD88						
NFKB1 (p50)						
NFKB2 (p100)						

NFKBIA (IκBα)						
NFKBIE (IκBε)	G34E +/-<br>probably damaging 0.978	G34E +/-<br>probably damaging 0.978	S33F +/-<br>possibly damaging 0.940<br>V194A +/-<br>benign 0.000<br>P175L +/-<br>benign 0.000	S33F +/-<br>possibly damaging 0.940<br>V194A +/-<br>benign 0.000<br>P175L +/-<br>benign 0.000	S33F +/-<br>possibly damaging 0.940<br>V194A +/-<br>benign 0.000<br>P175L +/-<br>benign 0.000	
PLCG1 (PLCγ1)	S279G +/-<br>benign 0.000<br>I813T +/-<br>benign 0.000	S279G +/-<br>benign 0.000<br>I813T +/-<br>benign 0.000				I813T +/-<br>benign 0.000
PLCG2 (PLCγ2)						
PRKCA (PKC)	V568I +/+<br>benign 0.000	V568I +/+<br>benign 0.000	V568I +/+<br>benign 0.000	V568I +/+<br>benign 0.000	V568I +/+<br>benign 0.000	V568I +/+<br>benign 0.000
PRKCB (PKC)						
PRKCZ (PKC)			S148R +/-<br>benign 0.002	S148R +/-<br>benign 0.002	S148R +/-<br>benign 0.002	
PRKCG (PKC)						
PRKCE (PKC)						
PRKCD (PKC)						
PRKCH (PKC)						
PRKCI (PKC)						
RELA (p65)						
RELB						
RIPK1 (RIP1)			A569V +/-<br>possibly damaging 0.617	A569V +/-<br>possibly damaging 0.617	A569V +/-<br>possibly damaging 0.617	
SYK						
TAB1						
TAB2						
TAB3	W394R +/+<br>benign 0.000	W394R +/+<br>benign 0.000	W394R +/+<br>benign 0.000	W394R +/+<br>benign 0.000	W394R +/+<br>benign 0.000	W394R +/+<br>benign 0.000
TICAM1 (TRIF)						
TIRAP			S180L +/+<br>possibly damaging 0.456	S180L +/+<br>possibly damaging 0.456	S180L +/+<br>possibly damaging 0.456	S180L +/-<br>possibly damaging 0.456

TLR4			D299G +/- benign 0.026 T399I +/- benign 0.177	D299G +/- benign 0.026 T399I +/- benign 0.177	D299G +/- benign 0.026 T399I +/- benign 0.177	P202T +/- possibly damaging 0.488
TNF						
TNFRSF1A (TNFR1)						
TNFRSF11A (RANK)	A192V +/- benign 0.051	A192V +/- benign 0.051				A192V +/- benign 0.051
TNFRSF13B (BAFFR)						
TNFSF11 (RANKL)						
TNFSF13B (BAFF)						
TRADD						
TRAF2						
TRAF3						M129T +/- benign 0.000
TRAF5						
TRAF6						
TRAM						
TRIM25	P358L +/+ benign 0.050	P358L +/+ benign 0.050				P358L +/- benign 0.050
ZAP70						
